# Novel Target Exploration from Hypothetical Proteins of *Klebsiella pneumoniae* MGH 78578 Reveals a Protein Involved in Host-Pathogen Interaction

**DOI:** 10.3389/fcimb.2020.00109

**Published:** 2020-04-03

**Authors:** G. Pranavathiyani, Jyoti Prava, Athira C. Rajeev, Archana Pan

**Affiliations:** Centre for Bioinformatics, School of Life Sciences, Pondicherry University, Pondicherry, India

**Keywords:** *Klebsiella pneumoniae*, hypothetical protein, functional annotation, drug target, vaccine target, host–pathogen interaction

## Abstract

The opportunistic pathogen *Klebsiella pneumoniae* is a causative agent of several hospital-acquired infections. It has become resistant to a wide range of currently available antibiotics, leading to high mortality rates among patients; this has further led to a demand for novel therapeutic intervention to treat such infections. Using a series of *in silico* analyses, the present study aims to explore novel drug/vaccine candidates from the hypothetical proteins of *K. pneumoniae*. A total of 540 proteins were found to be hypothetical in this organism. Analysis of these 540 hypothetical proteins revealed 30 pathogen-specific proteins essential for pathogen survival. A motifs/domain family analysis, similarity search against known proteins, gene ontology, and protein–protein interaction analysis of the shortlisted 30 proteins led to functional assignment for 17 proteins. They were mainly cataloged as enzymes, lipoproteins, stress-induced proteins, transporters, and other proteins (viz., two-component proteins, skeletal proteins and toxins). Among the annotated proteins, 16 proteins, located in the cytoplasm, periplasm, and inner membrane, were considered as potential drug targets, and one extracellular protein was considered as a vaccine candidate. A druggability analysis indicated that the identified 17 drug/vaccine candidates were “novel”. Furthermore, a host–pathogen interaction analysis of these identified target candidates revealed a betaine/carnitine/choline transporters (BCCT) family protein showing interactions with five host proteins. Structure prediction and validation were carried out for this protein, which could aid in structure-based inhibitor design.

## Introduction

*Klebsiella pneumoniae* is a Gram-negative, encapsulated, non-motile bacterium belonging to the *Enterobacteriaceae* family. It is commonly present in soil, water, and animals, including humans. This organism is a part of normal gut flora in human where it does not cause any infection. However, in healthcare environments, it can colonize in medical devices (viz., ventilators and intravenous catheters) and opportunistically infect immunocompromised patients admitted in the intensive care unit (CDC.gov., 2020). Indeed, this bacterium causes several infections, such as urinary tract infection, bacteraemia, pneumonia, and liver abscesses in hospitalized patients (Chung, 2016). Patients infected with *Klebsiella* can transmit the pathogen via direct contact or indirectly through contaminated medical devices (CDC.gov., 2020). This opportunistic pathogen is able to form biofilms in various biotic and abiotic surfaces like other pathogenic bacteria, including *Pseudomonas aeruginosa, Escherichia coli*, and *Acinetobacter baumannii* (Vuotto et al., 2014; de Campos et al., 2016; Riquelme et al., 2018). The formation of biofilms assists the pathogen in withstanding the host defense mechanism and antimicrobial agents.

Several outbreaks of *K. pneumoniae* infections have been reported in hospital settings from different countries, including China, Israel, Poland, Italy, Colombia, and the United States (Kaye and Dhar, 2016; Ocampo et al., 2016; Baraniak et al., 2017; Krapp et al., 2018; Sotgiu et al., 2018; Liu and Guo, 2019). Reportedly, *K. pneumonia*e accounts for ~12% of all hospital-acquired pneumonia in the world (Ashurst and Dawson, 2019). In addition to the presence of the beta-lactamase enzyme, which makes *K. pneumoniae* antibiotic resistant, an alteration in the upregulation of efflux pumps is reportedly making this opportunistic pathogen resistant to multiple drugs, including the last-resort treatment regimen carbapenems. This leads to high mortality rates among the patients (~50%) (Xu et al., 2017). According to the Center for Disease Control and Prevention (CDC), ~80% of the reported carbapenem-resistant Enterobacteriaceae infections in 2013 were due to *K. pneumoniae* (Ashurst and Dawson, 2019). Thus, the present scenario demands the development of novel therapeutic intervention for treating such bacterial infections.

In many organisms, the molecular functions of more than 30% proteins are unknown; these proteins are termed as “hypothetical proteins”. The functional annotation of hypothetical proteins can enable us to understand their roles in different metabolisms as well as to identify previously unexplored drug targets in an organism (Shahbaaz et al., 2016). Several bioinformatics resources, such as databases and tools, are available for functional annotation of hypothetical proteins. These resources have been successfully used to annotate the functions of hypothetical proteins in different bacterial pathogens, including *Borrelia burgdorferi* (Hassan et al., 2016), *Chlamydia trachomatis* (Turab Naqvi et al., 2017), *Helicobacter pylori* (Naqvi et al., 2016), *Haemophilus influenzae* (Shahbaaz et al., 2013)*, Mycobacterium tuberculosis* (Yang et al., 2019)*, Vibrio cholerae* (Islam et al., 2015), and *Staphylococcus aureus N315* (Prava et al., 2018). Out of the available proteome of *K. pneumoniae* MGH 78578, ~11% is made up of HPs, which can be potential resources to be studied both functionally and structurally. Although bioinformatics studies on a few hypothetical proteins, such as KPN_00953(YcbK) (Teh et al., 2014), KPN_02809 (a Zinc-Dependent Metalloprotease) (Wong et al., 2012), and KPN_00728, KPN_00729 (Chain C and D of Succinate Dehydrogenase, respectively) (Choi et al., 2009), are available, mining and analysis of all hypothetical proteins to shortlist drug/vaccine targets in this pathogen is yet unexplored. In the present study, a series of *in silico* analyses of 540 hypothetical proteins encoded by the *K. pneumoniae* genome were carried out to explore novel drug/vaccine candidates in this organism. The annotated target proteins can be further utilized to design and develop novel inhibitors for the treatment of Klebsiella infections.

## Materials and Methods

### Sequence Retrieval

The whole proteome of *K. pneumoniae* subsp. pneumoniae MGH 78578 (NC_009648.1) was retrieved from the National Center for Biotechnology Information (NCBI), a comprehensive web portal providing access to genomic and biomedical information (Sayers et al., 2019). The hypothetical proteins (HPs) from the whole proteome of *K. pneumoniae* were obtained using an in-house Perl script (https://gist.github.com/pranavathiyani).

### Essentiality and Non-homology Analysis

The collected HPs of *K. pneumoniae* were subjected to a similarity search using protein BLAST (BLASTp) (Altschul, 1990) against the essential proteins of bacteria present in the database of essential genes (DEG 15.2) with an *e*-value ≤ 0.0001 and bit-score ≥100 as cut-off (Jadhav et al., 2014). DEG is a repository of experimentally determined essential genes of various bacteria, archaea, and eukaryotes (Luo et al., 2014). The query genes/proteins that showed a similarity with genes/proteins of DEG were regarded as possible essential genes or proteins. The HPs that had at least one hit in DEG-BLAST were considered as essential proteins in the current study. Furthermore, a similarity search using BLASTp was carried out between identified essential proteins and the human proteome with a cut-off *e*-value ≥0.0001 (Jadhav et al., 2014). The essential pathogen proteins that showed no hit with the human proteome were considered as non-homologous to human proteins and used for further analysis.

### Function Prediction

Function prediction of essential non-homologous (ENH) proteins of *K. pneumoniae*, which resulted from the previous analysis, was carried out using bioinformatics resources like InterPro (Mitchell et al., 2019), Pfam (Finn et al., 2016), and NCBI-BLASTp (Altschul, 1990). The ENH protein sequences were submitted to InterPro and Pfam with default parameters to identify their motifs/domain families. InterPro is an integrated online resource of protein databases that provides detailed information about the protein families, domain, and motifs. Pfam uses a Hidden Markov Model-based method to identify the domain families of the proteins. Protein BLAST (BLASTp) search was performed for the ENH proteins to identify homologous sequences with known functions from the NCBI protein database (non-redundant). Moreover, the predicted function of the ENH proteins from InterPro, Pfam, and NCBI-BLASTp search was cross-checked with the function of DEG-hits obtained from an essentiality analysis. The predicted function was also compared and verified using gene ontology (GO) analysis and a protein–protein interaction analysis. The GO analysis was performed by submitting the annotated ENH proteins to CELLO2GO and GO FEAT. CELLO2GO is a web server that performs a similarity search (BLAST) for a given protein sequence to obtain its homologous sequences with GO annotation (molecular function, biological process, and cellular location) (Yu et al., 2014). GO FEAT is an online platform for functional annotation for genomic as well as transcriptomic data based on similarity search (Araujo et al., 2018). Furthermore, the ENH proteins were given as query to STRING database (version 11.0) with medium confidence (0.40) in order to identify functions based on the homolog hits and the interactions among the proteins. STRING database is an integrated resource of experimental and predicted protein-protein interactions (PPI). Currently, STRING comprises more than 2,000 million interactions of 24.6 million proteins from 5,090 organisms (Szklarczyk et al., 2019).

### Physicochemical Characterization and Virulence Prediction

The physicochemical properties, such as molecular weight, theoretical isoelectric point (pI), instability index, aliphatic index, and grand average of hydropathicity (GRAVY), of the annotated ENH proteins were computed using the ProtParam tool of Expasy. Expasy is a Swiss Institute of Bioinformatics resource portal for tools and databases used in diverse areas of life sciences, including genomics, proteomics, genetics, systems biology, molecular evolution, and transcriptomics (Gasteiger et al., 2005). Virulence proteins among the annotated ENH proteins were identified using a similarity search against the core dataset of virulent proteins from Virulence Factor Database (VFDB) with a cut-off *e*-value ≤ 0.0001. VFDB is a database for bacterial virulence factors primarily curated from the scientific literature (Chen et al., 2016). The annotated ENH proteins were submitted to VICMpred, an SVM-based functional classification server for Gram-negative bacterial proteins that functionally classify the proteins into different categories based on amino acid composition (Saha and Raghava, 2006). Further, an MP3 tool was used for the identification of pathogenic proteins among the functionally annotated ENH proteins. The MP3 tool utilizes an integrated approach based on SVM-HMM in order to better provide efficiency and accuracy in predicting pathogenic proteins (Gupta et al., 2014).

### Druggability and Subcellular Localization Analysis

To assess the druggability of the annotated ENH proteins, DrugBank and ChEMBL databases were utilized. DrugBank is a comprehensive bioinformatics database for cheminformatics comprising detailed information about drugs and the corresponding targets (Wishart et al., 2018). ChEMBL is a curated database of bioactive chemical compounds maintained by the EMBL. It includes manually curated data from the scientific literature on drug-like compounds along with their bioactivity determined based on assays (Gaulton et al., 2012). A similarity search was performed between the annotated ENH proteins and known targets of DrugBank and ChEMBL with a cut-off *e*-value ≤ 0.00001. Subcellular localization of the proteins was predicted using CELLO (v.2.5), a multi-class SVM-based classification server which uses amino acid sequence features for predicting subcellular localization. In the case of Gram-negative bacteria, the average accuracy of CELLO in localization prediction is 89% (Chen et al., 2006).

### Prediction of Host-Pathogen Interactions

The host–pathogen interactions of the annotated ENH proteins were predicted using the interlog method. This method relies on a homology search of the query sequence against the known host–pathogen interaction data. If the pathogen protein “A” interacts with host protein, “B” and if a protein “X” is homologous to protein “A”, then there is a high chance that protein “X” will also interact with protein “B” (Yu et al., 2004). Based on this principle, a homology search of the annotated ENH proteins was performed against the full database of HPIDB with default parameters (identify >50%, query coverage >50% and *e*-value = 0.00001) to obtain the proteins that interact with the human host. HPIDB 3.0 is a comprehensive database of curated host–pathogen interaction data (Kumar and Nanduri, 2010).

### Structure Prediction

The annotated ENH proteins were searched against the Protein Data Bank (PDB) using PSI-BLAST (Altschul, 1990) from NCBI. PDB is a repository of three-dimensional (3D) structure data for different biological macromolecules (Berman et al., 2000). Determining the 3D structure of the protein is important for understanding its molecular function at the atomic level and it also facilitates the process of structure-based drug design. Herein, the structure prediction for the selected protein was carried out using an I-TASSER server (Yang and Zhang, 2015) owing to the low structural similarity of the available 3D structure. The predicted structure was validated using SAVES server (https://servicesn.mbi.ucla.edu/SAVES/). The final 3D structure was visualized using the Pymol tool (version 2.3.3 Schrödinger, LLC).

## Results and Discussion

**Figure 1:**
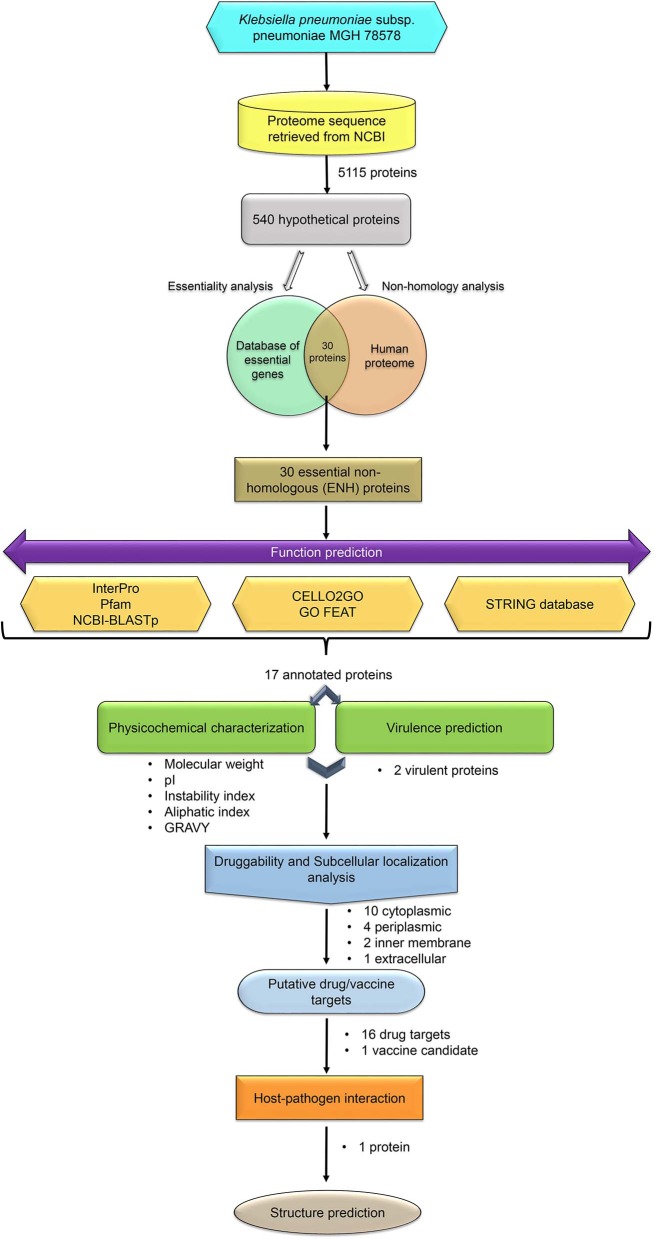
The complete workflow adopted in the present study.

In the present study, HPs encoded by the *K. pneumoniae* MGH 78578 genome were analyzed using the *in silico* approach to shortlist proteins that can be potential drug and vaccine targets. The workflow adopted in the current study is illustrated in Figure 1.

### Identification of Essential Pathogen-Specific Proteins

The complete genome of *K. pneumoniae* subsp. pneumoniae MGH 78578 is a single circular chromosome of 5.31 Mb in size. The genome comprises 5,115 protein-coding genes. The corresponding protein sequences were retrieved for the analysis. Out of 5,115 proteins, 540 were found to be HPs in this organism (Supplementary File 1) (at the time of retrieval). The identified HPs were subjected to essentiality and non-homology analysis in order to find out essential pathogen-specific proteins. The idea behind the essentiality analysis is to shortlist proteins that are crucial for the survival of the pathogen, and targeting those proteins would thus be lethal for the pathogen. The non-homology analysis will result in proteins that are exclusively present in the pathogen and absent in the human host. This approach can aid in designing drugs that target only pathogen proteins without interfering host system, thereby minimizing side-effects.

The present study utilized experimentally determined essential proteins of 48 bacterial species deposited in DEG to predict essential proteins among *K. pneumoniae* HPs. It is believed that the HPs of bacteria that are similar to known essential proteins of DEG are possible essential proteins. Out of 540 HPs, 40 were found to be similar to known essential proteins and were thus considered as possible essential proteins of this pathogen. Subsequently, the non-homology analysis of the 40 essential proteins revealed that 30 proteins did not have any hit with the human proteome, and they were therefore non-homologous to humans. These 30 essential non-homologous (ENH) proteins (Supplementary File 2) are essential for the survival of the pathogen and are pathogen-specific (present in the pathogen but absent in the human). Theoretically, all the shortlisted ENH proteins have the potential to become good drug/vaccine target(s) in *K. pneumoniae*. However, insight into the functional annotation, physicochemical properties, virulence, druggability, and subcellular localization of these 30 shortlisted proteins would provide an additional layer of refinement for target identification, and those analyses were thus performed subsequently.

### Function Prediction of ENH Proteins

The function prediction of 30 ENH proteins using bioinformatics secondary databases and tools, such as InterPro, Pfam, and NCBI BLASTp yielded 17 annotated proteins (Table 1) along with 10 uncharacterized domain proteins with unknown functions and three proteins with no hits/similarity (Supplementary File 3). It is worth mentioning at this point that our previous work on functional annotation of essential HPs from *Staphylococcus aureus* N315 assessed the performance of these tools using receiver operating characteristic (ROC) curve analysis and was noticed to have annotated the function of HPs with high accuracy (Prava et al., 2018). In the present study, the majority of the 17 annotated proteins fell into the category of enzymes, lipoproteins, transporters, and stress-induced proteins among others. The remaining 10 ENH proteins either belonged to uncharacterized domain(s)/family proteins (YaeP family protein, YccJ like protein, YfdX like protein) or different families of hypothetical proteins (YheO like PAS domain protein). The previous studies of these uncharacterized proteins have suggested their essentiality in bacteria, and some are reported to have condition-based expression in several organisms (Goodacre et al., 2014). For instance, the YfdX protein, which has been reported to be present in several bacterial pathogens, including *Escherichia coli, Salmonella* enterica serovar Typhi, and Typhimurium, is under the control of a regulator protein, EvgA, responsible for environmental stress expressions (McClelland et al., 2001; Masuda and Church, 2002). A recent study on Salmonella infection has demonstrated that YfdX is involved in virulence, antibiotic susceptibility, and in modulating pathogens' growth/survival strategies (Lee et al., 2019). The hypothetical protein WP_002920130.1 with a YheO-like PAS domain was predicted to have putative DNA binding activity apart from its transcription regulatory activity (InterPro entry: IPR013559).

**Table 1:** List of annotated essential non-homologous proteins and their predicted functions using InterPro, Pfam, and NCBI-BLASTp.

**Sl. No**	**Protein ID**	**InterPro**	**Pfam**		**NCBI BLASTp**
			**Identifier**	**Description**	
1	WP_002888808.1	Chaperone lipoprotein, PulS/OutS-like (IPR019114)	T2SS_PulS_OutS	Type II secretion system pilotin lipoprotein (PulS_OutS)	Chaperone lipoprotein YacC
2	WP_004222859.1	Endopeptidase, NLPC/P60 domain (IPR000064)	NLPC_P60	NlpC/P60 family	Lipoprotein NlpC/P60 family protein
3	WP_002890061.1	PapD-like superfamily (IPR008962)	PapD_N	Pili and flagellar-assembly chaperone, PapD N-terminal domain	Pilus assembly protein
4	WP_002890284.1	Pyrimidine/purine nucleoside phosphorylase (IPR009664)	DUF1255	Protein of unknown function (DUF1255)	Pyrimidine/purine nucleoside phosphorylase
5	WP_004151327.1	Pheromone shutdown, TraB (IPR002816)	TraB	TraB family	Conjugal transfer protein TraB
6	WP_012068456.1	YbfN-like lipoprotein (IPR025727)	YbfN	YbfN-like lipoprotein	Lipoprotein ybfN
7	WP_004176857.1	Cyclophilin-like domain superfamily (IPR029000)	CT_C_D	Carboxyltransferase domain, subdomain C and D	5-oxoprolinase subunit PxpB
8	WP_023288894.1	–	YmgB	Biofilm development protein YmgB/AriR	Two-component-system connector protein YcgZ
9	WP_073549749.1	BCCT transporter family (IPR000060)	BCCT	BCCT, betaine/carnitine/choline family transporter	BCCT family transporter
10	WP_002898708.1	Stress-induced protein, KGG, repeat (IPR019626)	KGG	Stress-induced bacterial acidophilic repeat motif	Stress-induced protein
11	WP_004150795.1	Stress-induced protein, KGG, repeat (IPR019626)	KGG	Stress-induced bacterial acidophilic repeat motif	Stress-induced acidophilic repeat motif-containing protein
12	WP_041937616.1	Putative bacterial toxin ydaT superfamily (IPR037042)	YdaT_toxin	Putative bacterial toxin ydaT	Bacteriophage regulatory protein CII
13	WP_004143718.1	Stress-induced protein, KGG, repeat (IPR019626)	KGG	Stress-induced bacterial acidophilic repeat motif	Stress-induced acidophilic repeat motif-containing protein
14	WP_002911528.1	Sugar-phosphate isomerase, RpiB/LacA/LacB family (IPR003500)	LacAB_rpiB	Ribose/Galactose Isomerase	Galactose-6-phosphate isomerase LacB subunit
15	WP_002914983.1	Phosphotransferase system, lactose/cellobiose-type IIA subunit (IPR003188)	PTS_IIA	PTS system, Lactose/Cellobiose specific IIA subunit	PTS system cellobiose-specific transporter subunit IIA
16	WP_002918629.1	Barstar-like superfamily (IPR035905)	Barstar	Barstar (barnase inhibitor)	barnase inhibitor
17	WP_015959101.1	Bactofilin A/B (IPR007607)	Bactofilin	Polymer-forming cytoskeletal	Ccm domain-containing protein

Predicted functions of 17 proteins were verified by a DEG-BLAST search that yielded similar function annotations in other organisms, thereby supporting our results. Of the 17 annotated proteins, eight proteins were found to have orthologs in *Escherichia coli*, a closely related organism of *Klebsiella*; orthologs of the remaining annotated proteins were found to be present in other bacterial species, including *Mycoplasma pulmonis, Salmonella, Pseudomonas aeruginosa*, and *Haemophilus influenzae* (Supplementary File 4). A GO analysis of the annotated 17 proteins revealed that these proteins were involved in various molecular functions, namely, peptidase activity, hydrolase activity, isomerase activity, enzyme regulator activity, ion binding, transmembrane transporter activity, and kinase activity (Supplementary File 5). This is in accordance with the function prediction results obtained from InterPro, Pfam, and NCBI BLASTp searches, signifying the reliability of our annotation. Catabolic processes, protein folding, cell differentiation, transport, response to stress, and small molecule metabolic processes were the major biological processes in which these annotated proteins were found to be involved. In addition, the PPI analysis of the 17 annotated proteins mapped 14 proteins in the interaction data, and, of these, 11 proteins were found to have annotated functions that are consistent with our function prediction results (Supplementary File 6). In the PPI network, it was observed that the PTS system protein (cellobiose-specific IIA component) had interacted with 4-deoxy-L-threo-5-hexosulose-uronate ketol-isomerase, and the secretion protein (chaperone lipoprotein YacC) had interacted with the TraB protein. The detailed discussion on the predicted molecular functions of the 17 annotated ENH proteins can be found in the subsequent section under different functional categories.

### Enzymes

Enzymes are a class of proteins that are involved in biochemical reactions in the form of catalysts to convert the substrate(s) to product(s). Among 17 annotated ENH proteins, four belong to different enzyme classes. For instance, the protein WP_002890284.1 was found to be involved in pyrimidine/purine nucleoside phosphorylase activity. In the nucleoside salvage pathway, phosphorylase enzymes participate in catalyzing the reversible phosphorolytic cleavage of the glycosidic bond of pyrimidine/purine nucleosides. They also play an important role in activating prodrugs (as analogs) and as inhibitors for antiparasitic and anticancer agents (Bzowska, 2015). The protein WP_004176857.1 was found to harbor a cyclophilin-like domain, which is involved in cyclosporine A (an immunosuppressive drug) binding. The proteins belonging to this family comprise of a beta-barrel domain, which is the core of cyclophilin-type peptidyl-prolyl cis-trans isomerases activity. This domain speeds up the protein folding by catalyzing the cis-trans isomerization of prolineimidic peptide bonds in oligopeptides (Takahashi et al., 1989). The protein WP_002911528.1 is a sugar phosphate isomerase, which includes sugar isomerases like ribose 5-phosphate isomerase B (RpiB), galactose isomerase subunit A (LacA), and galactose isomerase subunit B (LacB). The enzyme galactose-6-phosphate isomerase is induced by the presence of galactose or lactose in the cell. RpiB has a Rossmann-type alpha/beta/alpha sandwich topology and forms a homodimer (Takahashi et al., 1989; Zhang et al., 2003b). This protein is involved in catalyzing the interconversion of D-ribose 5-phosphate and D-ribulose 5-phosphate in the non-oxidative branch of the pentose phosphate pathway (Zhang et al., 2003a). The protein WP_002914983.1 was identified to be involved in the phosphoenolpyruvate-dependent sugar phosphotransferase system (PTS). The role of PTS is to serve as a transport system for carbohydrate in bacteria. It takes part in catalyzing the phosphorylation of incoming sugar substrates along with its translocation across the cell membrane, making PTS a link between the uptake and metabolism of sugars (Postma et al., 1993).

### Lipoproteins

Bacterial lipoproteins are a group of membrane proteins that are involved in diverse functions, such as cellular physiology, cell division, virulence, adhesion to host cells, and virulence factor translocation. The proteins WP_002888808.1, WP_004222859.1, and WP_012068456.1 were found to be similar to the chaperone lipoprotein YacC, lipoprotein NlpC/P60 family protein, and lipoprotein YbfN family, respectively. Apart from the novel addition of protein YacC, the Chaperon lipoprotein family, PulS/OutS-like comprises of pullulanase secretion protein (PulS) *in K. pneumoniae* (UniProt ID: P20440), the lipoprotein OutS protein of *Erwinia chrysanthemi* (UniProt ID:Q01567), and a functionally uncharacterized type II secretion protein EtpO (UniProt ID: Q7BSV3) of *E. coli* O157: H7. Reportedly, PulS and OutS interaction facilitates the insertion of secretins into the outer membrane indicating its chaperone-like role in bacterial systems (InterPro entry: IPR019114). In various bacterial lineages, NlpC/P60 proteins categorically belong to the cell wall peptidases family, which hydrolyses the d-γ-glutamyl-meso-diaminopimelate or N-acetylmuramate-L-alanine linkage in the cell wall (Xu et al., 2009).

### Stress-Induced Proteins

The present study predicted three proteins (WP_002898708.1, WP_004150795.1, and WP_004143718.1) as stress-induced proteins with a highly conserved KGG repeat. In *E. coli*, YciG protein from yciGFE operon was reported to have a similar motif. The protein YciG, under the regulation of the general stress response controller RpoS, showed significant resistance to thermal and acid stress (Robbe-Saule et al., 2007).

### Transporters

Among 17 annotated proteins, two (WP_004151327.1 and WP_073549749.1) were predicted as transporters. The former protein was predicted as a conjugal transfer protein TraB. TraB consists of nucleotide-binding motifs, suggesting its potential energy-providing role in plasmid DNA/Tra protein transport (Chandler and Dunny, 2004). The later one was predicted as an inner-membrane metabolism molecule, and it showed similarity with BCCT family transporter protein (InterPro entry: IPR000060). BCCT represents Betaine/Carnitine/Choline Transporters that have 12 transmembrane regions and four conserved tryptophan in the central region. This protein family is specific to compounds that have quaternary nitrogen atoms (Ziegler et al., 2010).

### Toxin, Skeletal, and Two-Component System Proteins

The protein WP_041937616.1 belongs to a putative bacterial toxin YdaT superfamily, which corresponds to a toxin–antitoxin protein system. These genetic modules were found in plasmids as well as in the chromosomes, and they encode for a toxin and its cognate antidote. They are reported to be important in maintaining multi-resistant plasmids and in the evolution of antibiotic resistance (Yamaguchi and Inouye, 2011). There are several theories proposed to explain this plasmid stabilizing toxin–antitoxin systems based on evolution. Targeting the toxin or the anti-toxin would lead to accumulation of proteins, which can lead to lethality.

The bactofilin A/B (WP_015959101.1) family of proteins covers a diverse range of functional roles in cytoskeletal polymer formation, which is conserved among bacterial species. The unique subcellular distribution, and the dynamics of bactofilins in different bacterial species, suggests its roles as versatile structural elements adopting a range of cellular functions (Kühn et al., 2010).

WP_023288894.1 was predicted as YcgZ, a two-component-system connector protein that plays a major role in biofilm formation and in providing an additional input signal into the two-component signaling pathway. YcgZ is a substrate of Lon protease that regulates the expression of an outer membrane protein, OmpF which serves as a passive diffusion pore (Duval et al., 2017). Soo et al. (2011) has reported that YcgZ is associated with resistance against several antibiotics.

### Other Proteins

The protein WP_002918629.1 was predicted as a barstar-like superfamily protein. Barstar proteins are small single-chain proteins that tackle the lethal effect of the active barnase enzyme, which is an extracellular ribonuclease. Barstar inhibits the activity of this enzyme by sterically blocking the active site with a helix and adjacent loop segment (Hartley, 1988; Buckle et al., 1994). Another protein WP_002890061.1, which was identified to have PapD-like superfamily domain, acts as a chaperone protein during pili and flagellar assembly. This protein, reportedly found in several pathogenic bacteria, also helps in mediating host cell surface adhesion (Barnhart et al., 2000).

### Physicochemical Characterization and Virulence Prediction

In the present study, the molecular weight of the annotated 17 proteins was found to be ranging from 5,953.2 to 35,235.6 Da. The highest and the lowest theoretical isoelectric points (pIs) of the proteins were 10.15 and 4.28, respectively. Molecular weight and pI help in experimental setup for protein purification and crystallization procedures. The instability index measures the stability of the protein in a test tube. If the instability index of a protein is <40 then it is believed to be a stable protein (Gill and von Hippel, 1989). Among the annotated ENH proteins, the index of 11 proteins was found to be <40, and they are thus likely to be stable proteins. The aliphatic index of proteins is calculated on the basis of the number of aliphatic residues in the protein—the higher the value, the higher the thermostability (Ikai, 1980). The aliphatic index of the annotated ENH proteins varied from 9.84 to 109.31. The average GRAVY (Grand Average of Hydropathicity) for the ENH proteins was −0.45 with maximum 0.587 and minimum −1.738. The GRAVY is calculated based on the total hydropathy values of amino acids divided by the length of the protein. The calculated physicochemical properties of the annotated ENH proteins are provided in Supplementary File 7. All the calculated physicochemical properties of the annotated proteins could be useful for further experimental studies of these proteins.

A homology search against the core set of virulent proteins from VFDB resulted in the identification of two virulence proteins—WP_004222859.1 and WP_002890061.1—with comparable similarity to proteins from *Listeria monocytogenes* and *E. coli*, respectively. WP_004222859.1 was identified to be homologous to iap/cwhA which encodes P60 protein, a major extracellular virulence protein in *L. monocytogenes*. This protein has been reported to be involved in pathogen survival and host invasion (Cabanes et al., 2002). From the function prediction, it was found that this protein has an endopeptidase NlpC/P60 domain. The protein WP_002890061.1 was found to be similar to yagV/EcpE, a part of *E. coli* common pilus (ECP). ECP is an extracellular adhesive fiber found in both pathogenic and commensal strains, and is involved in biofilm formation and host cell recognition (Rendón et al., 2007). Targeting virulence factors of a pathogen would hinder the progress of pathogenesis.

VICMpred categorizes proteins into different classes: virulence factors, information molecule, cellular process, and metabolism molecule. It was observed from the prediction that, out of 17 annotated ENH proteins, seven proteins were involved in cellular process, eight were involved in metabolism, and two proteins were involved in information and storage. Furthermore, the MP3 server identified eight proteins as pathogenic and the remaining nine as non-pathogenic proteins. Two proteins (WP_004222859.1 and WP_002890061.1), which were identified as pathogenic by VFDB and MP3, were considered as virulence factors in the present study. The comprehensive results of the predictions are given in Table 2.

**Table 2:** List of annotated essential non-homologous proteins with their subcellular localization and virulence prediction.

**Sl. No**	**Protein ID**	**Localization**	**Virulence prediction**		
		**CELLO**	**VICMPred**	**MP3**	VFDB^*^
1	WP_002888808.1	Periplasmic	Metabolism molecule	Pathogenic	–
2	WP_004222859.1	Periplasmic	Metabolism molecule	Pathogenic	Virulent
3	WP_002890061.1	Periplasmic	Cellular process	Pathogenic	Virulent
4	WP_002890284.1	Cytoplasmic	Metabolism molecule	Pathogenic	–
5	WP_004151327.1	Inner membrane	Cellular process	Pathogenic	–
6	WP_012068456.1	Periplasmic	Information and storage	Pathogenic	–
7	WP_004176857.1	Cytoplasmic	Information and storage	Pathogenic	–
8	WP_023288894.1	Cytoplasmic	Cellular process	Non-pathogenic	–
9	WP_073549749.1	Inner membrane	Metabolism molecule	Pathogenic	–
10	WP_002898708.1	Cytoplasmic	Cellular process	Non-pathogenic	–
11	WP_004150795.1	Extracellular	Metabolism molecule	Non-pathogenic	–
12	WP_041937616.1	Cytoplasmic	Cellular process	Non-pathogenic	–
13	WP_004143718.1	Cytoplasmic	Metabolism molecule	Non-pathogenic	–
14	WP_002911528.1	Cytoplasmic	Cellular process	Non-pathogenic	–
15	WP_002914983.1	Cytoplasmic	Cellular process	Non-pathogenic	–
16	WP_002918629.1	Cytoplasmic	Metabolism molecule	Non-pathogenic	–
17	WP_015959101.1	Cytoplasmic	Metabolism molecule	Non-pathogenic	–

* *“–” indicates no significant hits found in VFDB*.

### Druggability and Subcellular Localization Analysis

Druggability prediction of ENH proteins using DrugBank and ChEMBL revealed that the proteins were not similar to the available known targets. Thus, they can be considered as “novel targets” which can be validated further experimentally.

Determining protein subcellular localization is vital for understanding the role of proteins in a cell, and it also aids in the process of drug discovery and delivery. The current study utilized the subcellular localization tool CELLO, which has high accuracy in predicting subcellular localization of proteins in Gram-negative bacteria. The tool CELLO predicted 10 proteins as cytoplasmic, four as periplasmic, two as inner membrane proteins, and one as an extracellular protein (Table 2). The proteins predicted in the cytoplasm, periplasm, and inner membrane can be considered as drug targets, and extracellular proteins can be considered as vaccine targets (Barh et al., 2011). Thus, in total, 16 drug targets and one vaccine candidate were identified from the analysis, and this can be further validated using an experimental study.

### Host–Pathogen Interactions

**Figure 2:**
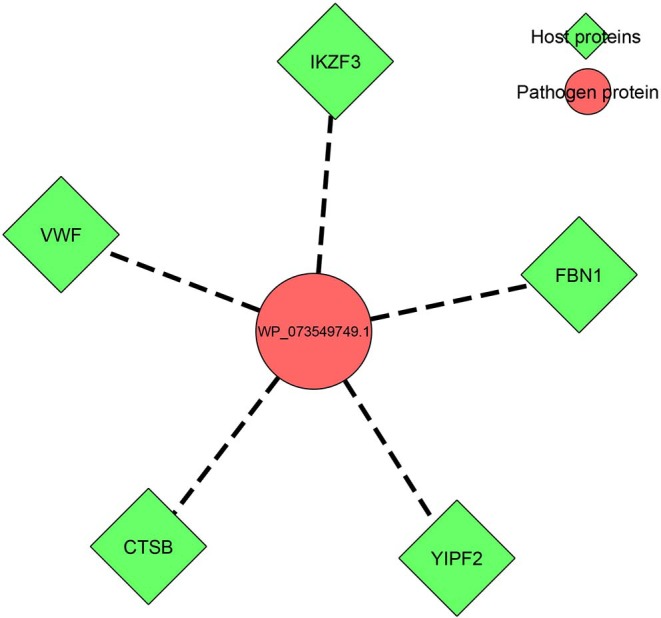
Host–pathogen interaction network of the identified target protein WP_073549749.1 (BCCT family) with five human host proteins.

Prediction of host–pathogen interactions among the hypothetical/annotated proteins in the organism could shed insight into understanding the pathogen biology, i.e., the pathogenesis as well as the host response, during infection/invasion. In the present study, from the 17 ENH proteins, the protein WP_073549749.1, which was annotated as a BCCT family transporter protein, was found to have interactions with five human host proteins, namely, von Willebrand factor, fibrillin-1, protein YIPF2, zinc finger protein Aiolos, and cathepsin B, as depicted in Figure 2. This prediction was based on an interlog method in which proteins similar to orthologs in interactions have an increased possibility of interacting with the same partner. The *K. pneumoniae* BCCT protein was found to be similar (75.6% sequence identity) to the putative quaternary ammonium transport protein (UniProt ID: Q8D0R9) from *Yersinia pestis*. The putative quaternary ammonium transport protein is encoded by beT2 gene, which reportedly has shown interactions with the abovementioned five human proteins (Supplementary File 8). The functions of the human proteins ranged from binding, catalytic activity, and transcription regulation to biological adhesion.

**Figure 3:**
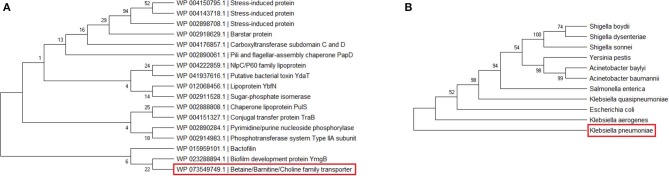
Phylogentic tree of **(A)** 17 annotated proteins and **(B)** *K. pneumoniae* BCCT protein and its orthologs.

Phylogenetic analysis of 17 annotated proteins was performed to find out interrelationship among these proteins. The analysis indicated that the BCCT protein, predicted to be involved in host–pathogen interaction, was grouped with biofilm-forming proteins, although they belong to diverse functional classes (Figure 3A). However, further study is needed to evaluate the relationship between these two proteins. Moreover, the orthologs of the *K. pneumoniae* BCCT protein were collected from UniProt using a BLASTp search, and a phylogenetic analysis was carried out for those BCCT proteins. It was observed that the *K. pneumoniae* BCCT protein was found to be closely related to *Klebsiella aerogenes* and *E. coli* (Figure 3B). Herein, the phylogenetic tree was built using maximum likelihood method with 500 bootstrap replicates in MEGA X, a molecular analysis tool for constructing phylogenetic tree (Kumar et al., 2018). Additionally, a phylogenetic tree was generated using PATRIC for 1,018 genomes belonging to the order Enterobacterales to have an insight into the evolutionary relationship of *K. pneumoniae* with the other members of *Enterobacteriaceae* family (Supplementary File 9). PATRIC is a bioinformatics resource platform that provides multi-omics data and analysis tools for biomedical research (Davis et al., 2020).

### Structure Prediction and Validation

**Figure 4:**
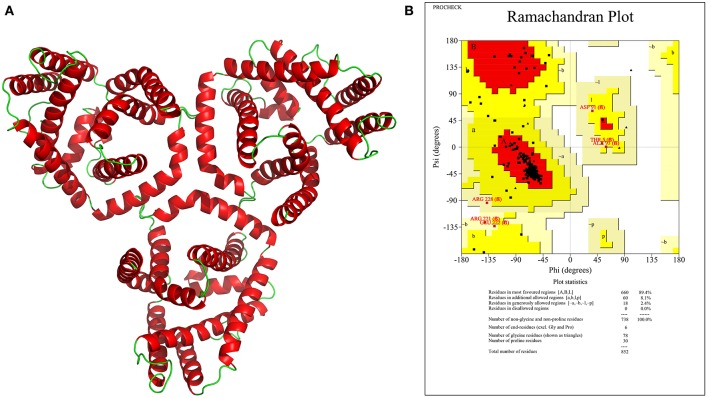
**(A)** Predicted 3D model structure of a BCCT family protein (WP_073549749.1) visualized in PyMol, **(B)** Ramachandran plot for the predicted model showing 89.4% of residues in most favored region.

Protein structure determination helps in understanding the functional domains responsible for its activity, which is invaluable for novel inhibitor development. The sequences with high similarity tend to have high structural similarity also; a similarity search of the identified 17 drug/vaccine targets against 3D structures of proteins from PDB was thus performed, which identified 10 homologous structures from *E. coli, S. enterica*, and *V. parahaemolyticus*. Sequence identity varied from 24.26 to 79.36% with seven proteins showing >30% identity (Table 3). Homologous structures can be used as a template for building 3D models for these proteins, which is underway in our laboratory. Here, however, we have reported the structure of an important host–pathogen interacting protein (WP_073549749.1) that showed interactions with the human host. Due to low structural similarity with the available 3D template, the protein structure was modeled using I-TASSER server. Among the five generated models, the best model was chosen based on the C-score (C-score = 1.24), which represents the confidence of the predicted model. The estimated TM-score and RMSD were 0.88 ± 0.07 and 3.8 ± 2.6 Å, respectively. The predicted 3D model of the BCCT protein was trimeric in nature comprising large helical structures. The model was validated using the SAVES server. It was observed that 89.4% of residues of the predicted structure were falling in the most favored regions in the Ramachandran plot while 8.1 and 2.1% were in the additionally and generously allowed region, respectively (Figure 4). This emphasized the validity of our model, and, additionally, molecular dynamics studies can be carried out using this model.

**Table 3:** List of identified 3D homolog structures of annotated ENH proteins.

**Sl. No**	**Protein ID**	**PSI-BLAST hit**	**Organism(s)**	**Identity (%)**	**Query coverage (%)**	***E* value**	**Score**	**PDB ID**
1	WP_002888808.1	–	–	–	–	–	–	–
2	WP_004222859.1	Chain A, Lipoprotein spr	*Escherichia coli* K-12	43.44	64	2.00E-31	111	2K1G_A
3	WP_002890061.1	Chain A, Probable Fimbrial Chaperone Ecpb	*Escherichia coli*	28.16	84	4.00E-13	67	5DFK_A
4	WP_002890284.1	Chain A, Upf0345 Protein Vpa0057	*Vibrio parahaemolyticus* RIMD 2210633	53.76	98	8.00E-28	97.4	2OYZ_A
5	WP_004151327.1	–	–	–	–	–	–	–
6	WP_012068456.1	–	–	–	–	–	–	–
7	WP_004176857.1	Chain B, Ybgj	*Escherichia coli* K-12	79.36	100	6.00E-126	355	5DUD_B
8	WP_023288894.1	–	–	–	–	–	–	–
9	WP_073549749.1	Chain A, Crystal Structure Of Carnitine Transporter	*Escherichia coli* K-12	26.67	89	7.00E-38	141	3HFX_A
10	WP_002898708.1	–	–	–	–	–	–	–
11	WP_004150795.1	–	–	–	–	–	–	–
12	WP_041937616.1	Chain A, Crystal Structure Of An Uncharacterized Protein Encoded By Cryptic Prophage	*Escherichia coli* O6	30.23	44	0.002	37.7	3C4R_A
13	WP_004143718.1	–	–	–	–	–	–	–
14	WP_002911528.1	Chain A, Crystal Structure Of Ribose-5-Phosphate Isomerase Lacab_rpib From Vibrio Parahaemolyticus	*Vibrio parahaemolyticus* RIMD 2210633	69.52	99	1.00E-104	301	3ONO_A
15	WP_002914983.1	Chain A, PTS system, N, N′-DiacetylchitobiosE-specific IIA component	*Escherichia coli*	64.58	92	1.00E-36	120	1WCR_A
16	WP_002918629.1	Chain A, Putative Cytoplasmic Protein	*Salmonella enterica* subsp. enterica serovar Typhimurium str. LT2	70	98	1.00E-42	134	5F4C_A
17	WP_015959101.1	Chain A, Bactofilin A	*Caulobacter vibrioides* CB15	24.26	60	2.00E-06	47.4	2N3D_A

## Conclusion

Understanding the functions of hypothetical proteins is important since it facilitates the further comprehension of their role in biochemical/physiological pathways and the identification of novel classes of therapeutic targets. The present study utilized an *in silico* approach to identify drug and vaccine targets from hypothetical proteins of *K. pneumoniae*. The study first predicted 30 pathogen-specific essential proteins, for which a functional analysis was carried out. The methodology utilized herein was enabled the annotation of the functions of hypothetical proteins with high confidence. It was found that the proteins have various functional roles as enzymes, lipoproteins, stress-induced proteins, and virulent proteins. However, the functional annotation for some of the proteins was not possible owing to insufficient information. Subcellular localization analysis revealed 16 proteins as drug targets (cytoplasmic, inner membrane, and periplasmic proteins) and one extracellular protein as a vaccine candidate. Structure prediction of one protein which was predicted to be involved in host–pathogen interaction is reported in this study and can be utilized for further experimental studies in this area. In addition, the structural analyses of identified target proteins and screening of potential inhibitors are underway in our laboratory.

## Data Availability Statement

The datasets generated for this study can be found in the article/Supplementary Material.

## Author Contributions

GP and AP conceived and designed the study. GP performed the experiment and analyzed the results. GP, AP, JP, and AR critically reviewed the analysis and contributed to the preparation of the final version of the manuscript.

### Conflict of Interest

The authors declare that the research was conducted in the absence of any commercial or financial relationships that could be construed as a potential conflict of interest.

## Supplementary Material

Supplementary File 1 List of all hypothetical proteins from *Klebsiella pneumoniae* considered in the study.

Supplementary File 2 List of shortlisted essential non-homologous proteins.

Supplementary File 3 List of identified uncharacterized domain family essential non-homologous (ENH) proteins.

Supplementary File 4 Identified essential proteins and the respective homologs from DEG.

Supplementary File 5 Identified essential non-homologous proteins along with predicted GO using CELLO2GO and GO_FEAT.

Supplementary File 6 Mapped ENH proteins on STRING PPI data.

Supplementary File 7 Calculated physicochemical properties of the identified essential non-homologous proteins.

Supplementary File 8 List of host proteins interacting with a BCCT family protein.

Supplementary File 9 Phylogenetic tree of 1,018 genomes from the order Enterobacterales generated using PATRIC.

## Abbreviations

BCCT: Betaine/Carnitine/Choline Transporters
BLAST: Basic Local Alignment Search Tool
CDC: Centers for Disease Control and Prevention
CELLO: subCELlular LOcalization predictor
CELLO2GO: subCELlular LOcalization prediction with functional Gene Ontology
DEG: Database of Essential Genes
ECP: *E. coli* common pilus
ENH: Essential Non-Homologous
GO: Gene Ontology
GRAVY: Grand Average of Hydropathicity
HMM: Hidden Markov Model
HPIDB: Host-Pathogen Interaction Database
HPs: Hypothetical Proteins
NCBI: National Center for Biotechnology Information
PATRIC: Pathosystems Resource Integration Center
PDB: Protein Data Bank
PPI: Protein-protein Interactions
PTS: Phosphotransferase System
SAVES: Structure Analysis and Verification Server
STRING: Search Tool for the Retrieval of Interacting Genes/Proteins
SVM: Support Vector Machine
VFDB: Virulence Factor Database.
