# Supplementary material for: Novel Target Exploration from Hypothetical Proteins of Klebsiella pneumoniae MGH 78578 Reveals a Protein Involved in Host-Pathogen Interaction
Source: Front Cell Infect Microbiol. 2020 Apr 3;10:109. doi: 10.3389/fcimb.2020.00109 (PMC7146069; doi:10.3389/fcimb.2020.00109)
Supplement: Supplementary File 2 — List of shortlisted essential non-homologous proteins. [file Data_Sheet_2.docx]

**Supplementary file 2**: List of shortlisted essential non-homologous proteins

>NC_009648.1_prot_WP_002888808.1_137 [locus_tag=KPN_RS00700] [protein=hypothetical protein] [protein_id=WP_002888808.1] [location=complement(155134..155481)] [gbkey=CDS]

MKTLFRTMVLGSLLALSANSYALSESEAEDMADLTAVFVFLKNDCGYQNLPNTQIRRALV

FFAQQNQWDLSNYDSYNMKALGEDSYRDLSGINIPTAKKCKALARDSLSLLAYVK

>NC_009648.1_prot_WP_002889277.1_181 [locus_tag=KPN_RS00935] [protein=hypothetical protein] [protein_id=WP_002889277.1] [location=complement(203775..204398)] [gbkey=CDS]

MLVYWLDIIGTAVFAISGVLLAGKLRMDPFGVLVLGVVTAVGGGTIRDMALANGPVFWVK

DPTDLVVAMVTSMLTILLVRQPRRLPKWILPVLDAVGLAVFVGIGVNKAFLAGSGPLVAV

CMGVVTGVGGGIIRDVLAREIPMILRTEIYATACIVGGIVHATAHDTFHLPLENSAMMGM

VVTLVIRLAAIRWHLKLPTFALDDNGR

>NC_009648.1_prot_WP_002889429.1_213 [locus_tag=KPN_RS01090] [protein=hypothetical protein] [protein_id=WP_002889429.1] [location=complement(239664..239864)] [gbkey=CDS]

MDHYCELIRKRYAEIASGDLGYIPDALGCVLKVLNEIAADEALSESVREKAAYAAANLLV

SDYVNE

>NC_009648.1_prot_WP_026005907.1_274 [locus_tag=KPN_RS01420] [protein=hypothetical protein] [protein_id=WP_026005907.1] [location=301860..302360] [gbkey=CDS]

MHYHQRALLIILCGLLCACMVLATQVYRGARRDTEEVNNLICTAKSNVYIKSRKLILSGT

LVLDLKSNRIAIHYAVQDQQREQRLFFQDITISAPNRIGPKTYTFRIEAVHKFDSDTTGE

MFSWLRLLQPATVNELTINKVGQRTYLFSLNRQIYNFCTTSGSTKA

>NC_009648.1_prot_WP_004183096.1_277 [locus_tag=KPN_RS28530] [protein=hypothetical protein] [protein_id=WP_004183096.1] [location=304745..305233] [gbkey=CDS]

MHHYQRTVLNSITLLILAVSFALLILHVRQQRYPLSDLNAFLCTTRTVTSVQPGNFHADG

NIVLDFKNKRITLQYDIITAQQIKKVLYRDVYIKNLKMPGAGIYTFDVDSVKVFSTDTAG

DFLAHFRLLHPGAANEIRVTRVGNSTYLYSINRQIYNVCTLQ

>NC_009648.1_prot_WP_004222859.1_295 [locus_tag=KPN_RS01520] [protein=hypothetical protein] [protein_id=WP_004222859.1] [location=complement(321074..321640)] [gbkey=CDS]

MKPKLTHALFLIPFLLLAGCSSSPKQAKNTKSHADMTIDGGSDDLIPVVAALHDQMHTWQ

GTPYEWGGTEQSGVDCSGFVWRTLKDRFNLPMERITTRELLHMGVRVNKRDLRPGDLVFF

RTRAGMHVGFYDTDHNFLHASSSQGVMRSSLDNPYWESAFYQARRLPKEYNAQITMNSDT

LHLAKNRR

>NC_009648.1_prot_WP_002890061.1_310 [locus_tag=KPN_RS01585] [protein=hypothetical protein] [protein_id=WP_002890061.1] [location=335550..336260] [gbkey=CDS]

MSRRRGATLTKALLTVGCLLAAPLAQAISVGNLTFSLPAEADFASKRVVNNNKSARLYRI

AVSAIDRPGGSEVRSRPVDGELLFAPRQLVLQAGESEYFKFYYHGPRDNRERYYRVSFRE

IPTRNLTRRSPTGGEVSMEPVVVMDTILVVRPREVQFKWSFDKVAGTVSNTGNTWFKLLI

KPGCDSTEEEGDAWYLRPGDVVRQPALRQPGNHYLVYNDKFIKISDTCPLKPRPAE

>NC_009648.1_prot_WP_002890284.1_350 [locus_tag=KPN_RS01795] [protein=hypothetical protein] [protein_id=WP_002890284.1] [location=373192..373476] [gbkey=CDS]

MLQSNEYFDGKVKSIGFTSSSTGRASVGVMAEGEYTFGTAQPEEMTVVSGALNVLLPGET

EWKVYAAGEVFNVPGNSEFHLQVAEPTSYLCRYL

>NC_009648.1_prot_WP_004151327.1_478 [locus_tag=KPN_RS02465] [protein=hypothetical protein] [protein_id=WP_004151327.1] [location=complement(510442..511236)] [gbkey=CDS]

MGLLNRIKMLWRAAVGSSYSWPAMDIVLPGERYLHLVGSIHMGTRDMAPPPAKLLKKIRQ

ADALIVEADISGNETPFSNLPTYPPLAERLSDAQLSELEARAGELGLSVGLFDSQPLWQV

AMVLQATQAQKLGLRPDYGIDYQLLMAARESNIAVMELEGADSQIALLRDLPDGGMALLE

DTLTHWRTNARLLQVMIGWWLEQPPSRGAVSLPTTFSQSLYDVLMHQRNLAWRETLLALP

PGRYVVAVGALHLYGEGNLPDMLT

>NC_009648.1_prot_562 [locus_tag=KPN_RS02895] [protein=hypothetical protein] [pseudo=true] [location=597635..598716] [gbkey=CDS]

MKYTLPALTLAISAALSGCATPHSSAVSQPVVDSPVPNVAQPLQRQLAEGLYEMALSPQG

DALYVASAEGFKNVQGGAVYTLDPHTLNTIGLTHTDLKNFALQLSAEGKTLYVSNSLDGG

ISAIDTATGKVKNRLLFSERNEKGRPYGARQLLLLNNTLYVGAVATRRKSGWLTPLP*S*

KRGLKIPVNG*PACTTPRKPVACTPLTAAVKFW*STRVTNALNSAGSRWATSRRCCLIWP

KIATPAVCS*PTTRKRKPLWCWISTAASCLNSSTSAIRWRCSSIRNATKFIFHSVNPARS

SALTPAATR*RKAGRCRPIPTACCFPPMVRRCLSPSNSPSIKTTPLKARTASYVST*TRN

>NC_009648.1_prot_WP_012068456.1_736 [locus_tag=KPN_RS03775] [protein=hypothetical protein] [protein_id=WP_012068456.1] [location=791744..792076] [gbkey=CDS]

MKKMLLIAMMAAGLVACTTSPAPKEDTKLKDAYSACINTAEGNPDKIEACQSVLNVLKKD

KQHQQFTNQESVRVLDYQQCIQATRTGNDQAVKARCDQIWKEIRSNNTTH

>NC_009648.1_prot_WP_004176857.1_755 [locus_tag=KPN_RS03870] [protein=hypothetical protein] [protein_id=WP_004176857.1] [location=810600..811256] [gbkey=CDS]

MQRARCYLLGETAVVLELEPPVTLESQKRIWGLTQRLTDREEVVEVIPGMNNITVVLRRP

QEMAWEAIDKLQRWWEESDALEPESREISIPVIYGGEAGPDLGDVARHSGLSEKQVVELH

SSVEYMVWFLGFQPGFPYFGGLPEQLAMPRRAEPRVLVPAGSVGIGGSQTGIYPLATPGG

WQLLGRTPLALFDPKREEPVLLRSGDRVRFVPQKEGVC

>NC_009648.1_prot_WP_023288894.1_815 [locus_tag=KPN_RS04205] [protein=hypothetical protein] [protein_id=WP_023288894.1] [location=complement(867963..868208)] [gbkey=CDS]

MQQNGYIPDTANAIAQYFNKASLPSQQETLGQIVMDILNEGRHLNRKALCTKLLSRLDRA

RAPEEESHYQTLIGLLFADQE

>NC_009648.1_prot_WP_073549749.1_1062 [locus_tag=KPN_RS05435] [protein=hypothetical protein] [protein_id=WP_073549749.1] [location=complement(1162773..1163735)] [gbkey=CDS]

MSALTGLPDNFTVQAFVILLSGGIFCMSSWIGINNGLQRLSKMVGWGAFLLPLVVLLVGP

TEFITNNVINAVGLTTQNFLQMSLFTDPLGDGAFTRNWTVFYWLWWISYTPGVAMFVTRV

SRGRKIKEVIWGLLLGSTAGCWFFFGVMESYAMHQFVNGVINVPQVMQTLGGETAVQQVL

MSLPAGKLFLAAYLFVMIVFLASHMDAVAYTMAATSTRNLREGEDPDRGMRLFWCVVITL

IPLSILFTGASLETMKTTVVLTALPFLAILLIKTGGFVRWLKQDYAHVPVHQIETHTPEP

IIKAETLPVGAVLKGDGQSL

>NC_009648.1_prot_WP_002898701.1_1076 [locus_tag=KPN_RS05505] [protein=hypothetical protein] [protein_id=WP_002898701.1] [location=complement(1177398..1177625)] [gbkey=CDS]

MPTQEAKAHRVGEWASLRNTSPEIAEAIFEVAHYDEKLAEQIWEEGSDEVLALAFAKTDK

DSLFWGEQTIERKNV

>NC_009648.1_prot_WP_002898708.1_1078 [locus_tag=KPN_RS05520] [protein=hypothetical protein] [protein_id=WP_002898708.1] [location=1178678..1178851] [gbkey=CDS]

MANHRGGSGNFAEDRERASEAGRKGGQHSGGNFKNDPQRASEAGKKGGKNSHGSRES

>NC_009648.1_prot_WP_002900775.1_1154 [locus_tag=KPN_RS05900] [protein=hypothetical protein] [protein_id=WP_002900775.1] [location=1253754..1254296] [gbkey=CDS]

MIIYLHGFDSNSPGNHEKVMQLQFIDPDVRLISYSTRHPKHDMQHLLKEVDKMLQLTADD

RPLICGVGLGGYWAERIGFLCDIRQAVFNPNLFPHENMEGKIDRPEEYADIATKCVTNFR

EKNRDRCLVVLSRQDEALDSQRSADLLHHYYEIIWDEEQTHKFKNISPHLQRLKAFKTLG

>NC_009648.1_prot_WP_004150795.1_1202 [locus_tag=KPN_RS06155] [protein=hypothetical protein] [protein_id=WP_004150795.1] [location=1304530..1304715] [gbkey=CDS]

MAEHRGGSGNFAEDREKASEAGRKGGQHSGGNFKNDPQRASEAGKKGGQNSHGGGRKSDN

S

>NC_009648.1_prot_WP_041937616.1_1455 [locus_tag=KPN_RS07445] [protein=hypothetical protein] [protein_id=WP_041937616.1] [location=1553475..1554011] [gbkey=CDS]

MHTLSFQQSTGFNAGALIKRYQPIVAEHDNIRSAVRAWAAAEGQDVVSAYIVDEWRQQGG

EEIAFPDDISRARQKLFRYLDNPAESERYREYVRLLTPAIMAVLPLEYRHRLLPVDSFMS

RLARLEKETSEAKVAVAMGAPRHQKLKELSEGIVEMFRIDPELTAPLMAIVTSMLGAL

>NC_009648.1_prot_WP_004176301.1_1639 [locus_tag=KPN_RS08330] [protein=hypothetical protein] [protein_id=WP_004176301.1] [location=1728013..1728369] [gbkey=CDS]

MELASLVRDILRQRHVERLAQHREQDNEAEAEWEIARWRHRSGVVIQRRYELEIAQQDSA

SCPECWIDWQVIDAAGQEINPMRKQFYNLCQQSFWLAMQAGGEDETSDRIAQITDNHP

>NC_009648.1_prot_WP_004143718.1_1956 [locus_tag=KPN_RS09915] [protein=hypothetical protein] [protein_id=WP_004143718.1] [location=complement(2030335..2030520)] [gbkey=CDS]

MAEHRGGSGNFAEDREKASEAGRKGGQHSGGNFKNDPERASEAGKKGGKNSHGGGRKSGD

S

>NC_009648.1_prot_WP_002911528.1_2552 [locus_tag=KPN_RS12945] [protein=hypothetical protein] [protein_id=WP_002911528.1] [location=complement(2627909..2628547)] [gbkey=CDS]

MKIALMMENSQANKNAIILKELNAVADEKGFPVYNVGMSDENDHHLTYIHLGIMASILLN

SKAVDFVVTGCGTGQGALMSLNIHPGVVCGYCIDPADAFLFAQINNGNALSLPFAKGFGW

GAELNVRFIFEKAFTGRNGEGYPPERKEPQVRNAGILNQVKAAVVKENYLDTLRAIDPQL

VKTAVSGPRFQQCFFENCQDKAIEDFVRQIVA

>NC_009648.1_prot_WP_002914983.1_3227 [locus_tag=KPN_RS16485] [protein=hypothetical protein] [protein_id=WP_002914983.1] [location=3373028..3373342] [gbkey=CDS]

MELEEQVMGIIINAGQSRSLCYEALHAAKAGDFATADAKMQEAAHYSREAHLVQTQLIEA

DEGEGKTKMTLVMVHAQDHLMTSILAKELIAELIAIYRAQPLHA

>NC_009648.1_prot_WP_002915104.1_3255 [locus_tag=KPN_RS16620] [protein=hypothetical protein] [protein_id=WP_002915104.1] [location=3399809..3400186] [gbkey=CDS]

MNPYLQEVLDAHVLIERWLSHGEGSAEALMKRFAADFTMIPLSGEKMDYPTVSRFFHHAG

GSRPGLDIVVDQMEIISEWHDGAAVLYRESQTLADSSQNVRWSTAIFQQAEGKIVWRHLQ

ETRLG

>NC_009648.1_prot_WP_041937675.1_3689 [locus_tag=KPN_RS18865] [protein=hypothetical protein] [protein_id=WP_041937675.1] [location=complement(3841453..3842085)] [gbkey=CDS]

MKKVILASLLATMMSTSPVWATDSATAAPAAAATTQVQKEAADVLQVAVQGANAMRDIQF

ARLALFHGQPDSAKKLTDDAAALLAADDASWAKFVKTDAKAKMIADRYVIINASIALSED

YVATPEKESAIQSANEKLAKGDQKGAIDTLRLAGIGVIENQYLMPLNQTRKAVAQSQELL

KAGKYYEANLVLKGAEEGIVVDSEMLVAGN

>NC_009648.1_prot_WP_002918223.1_3773 [locus_tag=KPN_RS19300] [protein=hypothetical protein] [protein_id=WP_002918223.1] [location=complement(3922932..3923375)] [gbkey=CDS]

MDTLAAIGRWLSKQHVVTWCVSREDELWCANAFYVYDPDTVAFYLLSEEHTRHGQMTGQR

AKVAGTVNGQPKTVALIRGVQFKGEIRRLSGDEEARMRQRYVKRFPVARMLSAPVWEIRP

DEIKFTDNTLGFGKKLHWRRDAGAEQA

>NC_009648.1_prot_WP_002918629.1_3861 [locus_tag=KPN_RS19745] [protein=hypothetical protein] [protein_id=WP_002918629.1] [location=complement(4006419..4006694)] [gbkey=CDS]

MTTYTFDFDEIAEQQDFYREFSRTFELAQDKVNNLDSLWDAVTGGLLPLPLDIEFIHLND

KQRRRFGALILLFDEAEEELEGELRFNARQA

>NC_009648.1_prot_WP_002920130.1_3944 [locus_tag=KPN_RS20165] [protein=hypothetical protein] [protein_id=WP_002920130.1] [location=complement(4075306..4076028)] [gbkey=CDS]

MSRSLLTNETSELDLLDQRPFDQTDFDILKSYEAVVDGLAMLIGSHCEIVLHSLQDLKCS

AIRIANGEHTGRQIGSPITDLALRMLHDMTGADSSVSKCYFTRAKSGVLMKSVTIAIRNR

DHRVIGLLCINMNLDVPFSQIMSTFIPPETPEVNSPVNFASSVDDLVAQTLEFTIEEVNA

DRSVSNNAKNRQIVLNLYEKGIFDIKDAINQVADRLNISKHTVYLYIRQFKSGDFQGLDK

>NC_009648.1_prot_WP_015959101.1_4024 [locus_tag=KPN_RS20560] [protein=hypothetical protein] [protein_id=WP_015959101.1] [location=4161128..4161781] [gbkey=CDS]

MDKQYLAINGALFFWLLALIAWSVDASALARLAAACALIAFLLHSQRNKINAMFIKKNKT

EPQISEAATPPAINPEPEAVASKKHETTVIASGVHFVGNIVASGHVYIHGQVTGNIEAKE

HLIKVMREGQVEGNVSCRELIIDGKVQGQCHGDSITIEEHGHLEGTLAYRALAIKKGGVF

SGRAELLAAAENKSHILGLVADAPSKADAEPVRPQSA

>NC_009648.1_prot_WP_004186821.1_4971 [locus_tag=KPN_RS25485] [protein=hypothetical protein] [protein_id=WP_004186821.1] [location=complement(5179832..5180356)] [gbkey=CDS]

MRFALLLLWLTILAPAAHAADWLTWRRVGEATLTWGPFTVYHSQLRTPNGRYDGPQQDRA

LIITYRRDIDREALVEATRDQWQAQGILQQEPRSEAWLRMLQGIWPDVAPGSQLAFVVSG

GEGQFWYRASAAQTAFTPLGPRQSAAFSTRFLAIWLDPRTTYPELRQQLIGGTP
