# Supplementary material for: Novel Target Exploration from Hypothetical Proteins of Klebsiella pneumoniae MGH 78578 Reveals a Protein Involved in Host-Pathogen Interaction
Source: Front Cell Infect Microbiol. 2020 Apr 3;10:109. doi: 10.3389/fcimb.2020.00109 (PMC7146069; doi:10.3389/fcimb.2020.00109)
Supplement: Supplementary File 3 — List of identified uncharacterized domain family essential non-homologous (ENH) proteins. [file Data_Sheet_3.docx]

**Supplementary file 3:** List of identified uncharacterized domain family essential non-homologous proteins

| Sl. No | **Protein ID** | **InterPro** | **Pfam HMMER** | | **NCBI BLASTp** |
| --- | --- | --- | --- | --- | --- |
|  |  |  | **Identifier** | **Description** |  |
| 1 | WP_002889277.1 | Uncharacterised domain UPF0126 (IPR005115) | UPF0126 | UPF0126 domain | inner membrane protein |
| 2 | WP_002889429.1 | Uncharacterised protein family UPF0253 (IPR009624) | UPF0253 | Uncharacterised protein family (UPF0253) | YaeP family protein |
| 3 | WP_026005907.1 | - | - | - | inner membrane/periplasmic protein |
| 4 | WP_004183096.1 | - | - | - | - |
| 5 | prot_562 | - | - | - | YncE family protein |
| 6 | WP_002898701.1 | YccJ-like protein (IPR025600) | YccJ | YccJ-like protein | yccJ-like family protein |
| 7 | WP_002900775.1 | Uncharacterised protein family UPF0227/Esterase YqiA (IPR008886) | UPF0227 | Uncharacterised protein family (UPF0227) | YcfP protein |
| 8 | WP_004176301.1 | - | - | - | - |
| 9 | WP_002915104.1 | Uncharacterised conserved protein UCP029394 (IPR016918) | - | - | DUF4440 domain-containing protein |
| 10 | WP_041937675.1 | Uncharacterised protein family YfdX (IPR021236) | YfdX | YfdX protein | yfdX |
| 11 | WP_002918223.1 | Uncharacterised protein family UPF0306 (IPR011194) | - | - | yhbP |
| 12 | WP_002920130.1 | YheO-like (IPR013559) | PAS_6 | YheO-like PAS domain | Putative DNA-binding protein/transcription regulator |
| 13 | WP_004186821.1 | - | - | - | Protein of uncharacterised function (DUF2878) |
