# Supplementary material for: Novel Target Exploration from Hypothetical Proteins of Klebsiella pneumoniae MGH 78578 Reveals a Protein Involved in Host-Pathogen Interaction
Source: Front Cell Infect Microbiol. 2020 Apr 3;10:109. doi: 10.3389/fcimb.2020.00109 (PMC7146069; doi:10.3389/fcimb.2020.00109)
Supplement: Supplementary File 5 — Identified essential non-homologous proteins along with predicted GO using CELLO2GO and GO_FEAT. [file Data_Sheet_5.docx]

**Supplementary file 5**: Identified essential non-homologous proteins along with predicted GO using CELLO2GO and GO_FEAT

| **Protein ID** | **CELLO2GO** | | | **GO_FEAT** |
| --- | --- | --- | --- | --- |
|  | **Molecular Functions** | **Biological Process** | **Cellular Component** |  |
| WP_002888808.1 |  |  |  |  |
| WP_004222859.1 | Peptidase activity; Hydrolase activity | Catabolic process; Cell wall organization or biogenesis | cell; plasma membrane; extracellular region; cell wall; external encapsulating structure | |
| WP_002890061.1 |  |  | cell; external encapsulating structure | GO:0030288 - outer membrane-bounded periplasmic space; GO:0071555 - cell wall organization |
| WP_002890284.1 |  |  |  | GO:0004731 - purine-nucleoside phosphorylase activity; GO:0004850 - uridine phosphorylase activity; GO:0009032 - thymidine phosphorylase activity; GO:0016154 - pyrimidine-nucleoside phosphorylase activity; GO:0047975 - guanosine phosphorylase activity |
| WP_004151327.1 |  |  |  |  |
| WP_012068456.1 |  |  | cell; plasma membrane | |
| WP_004176857.1 | isomerase activity; enzyme regulator activity; ion binding | Protein folding; Cellular protein modification process; Cell differentiation; anatomical structure formation involved in morphogenesis; anatomical structure development | cell; plasma membrane | |
| WP_023288894.1 |  |  |  |  |
| WP_073549749.1 |  | Transport; Cellular nitrogen compound metabolic process; small molecule metabolic process; response to stress | | GO:0005886 - plasma membrane; GO:0016021 - integral component of membrane; GO:0022857 - transmembrane transporter activity; GO:0071705 - nitrogen compound transport |
| WP_002898708.1 |  |  |  |  |
| WP_004150795.1 |  |  |  |  |
| WP_041937616.1 |  |  |  |  |
| WP_004143718.1 |  |  |  |  |
| WP_002911528.1 |  |  |  | GO:0004751 - ribose-5-phosphate isomerase activity; GO:0005975 - carbohydrate metabolic process |
| WP_002914983.1 | Transmembrane transporter activity; Kinase activity; ion binding | Transport | intracellular; cell; cytoplasm | GO:0008982 - protein-N(PI)-phosphohistidine-sugar phosphotransferase activity; GO:0009401 - phosphoenolpyruvate-dependent sugar phosphotransferase system; GO:0046872 - metal ion binding; GO:0103111 - D-glucosamine PTS permease activity |
| WP_002918629.1 |  |  |  |  |
| WP_015959101.1 |  |  |  | GO:0016021 - integral component of membrane |
