# Supplementary material for: Novel Target Exploration from Hypothetical Proteins of Klebsiella pneumoniae MGH 78578 Reveals a Protein Involved in Host-Pathogen Interaction
Source: Front Cell Infect Microbiol. 2020 Apr 3;10:109. doi: 10.3389/fcimb.2020.00109 (PMC7146069; doi:10.3389/fcimb.2020.00109)

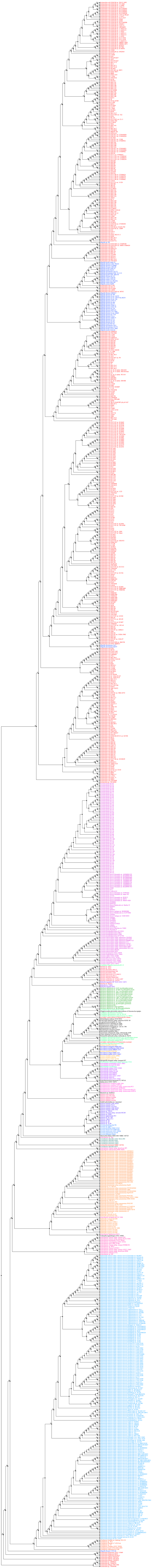

Supplement: Supplementary File 9 — Phylogenetic tree of 1,018 genomes from the order Enterobacterales generated using PATRIC. [file Data_Sheet_9.pdf]
